# The Chemical Metamorphosis of the Fluids of the Mouth with Relation to Discoloration of Gold

**Published:** 1891-12

**Authors:** Edward S. Niles


### International Dental Journal.

Vol. XII. December, 1891. No. 12.

### Original Communications.[1]

### THE CHEMICAL METAMORPHOSIS OF THE FLUIDS OF THE MOUTH WITH RELATION TO DISCOLORATION OF GOLD.[2]

BY EDWARD S. NILES, D.M.D.

The subject of chemical metamorphosis in the mouth is one that requires very long and deep scientific study. The particular phase that I want to present to-night is one perhaps of minor value in our profession, and yet, incidentally, it is of great importance.

Some time ago I was interested in the phosphates, and a suggestion presented itself to me while investigating the different compounds which I have always intended to follow out. Since that time various hints in the same line have come up which I have mentally pigeon-holed until the time when I should be able to confirm my impressions and present them to the profession.

The discoloration of gold in the mouth has never, to my mind, been satisfactorily explained, both with regard to that of fillings as well as plates, and I doubt if any one has really put his mind to work upon the subject to discover what really is the cause of the

[1] The editor and publishers are not responsible for the views of authors of papers published in this department, nor for any claim to novelty, or otherwise, that may be made by them. No papers will be received for this department that have appeared in any other journal published in the country.

[2] Read before the Harvard Odontological Society, May 28, 1891.

trouble. As I call your attention this evening to certain facts, it will seem very plain at least that previous opinions are not at all well founded.

First, you know that the gold which we use for filling must be pure gold, containing no alloy whatever,—at least I am told by Dr. Watts, one of our oldest manufacturers of gold-filling materials, that the least alloy will destroy its cohesive quality. Dr. Watts smiled when I asked him what metals were used to alloy the gold used for filling, and said, "If you only knew that a slight trace of alloy would render it non-cohesive you would know that all gold used for fillings must be essentially pure."

In the lectures at the school Dr. T. H. Chandler gives it as his opinion that in manipulating by mechanical contact with the iron from our instruments, or in burnishing, we rubbed off some portions of metal on to the gold. But if you stop to think of this idea a moment you will see how improbable it is. The gold is soft, while the instrument is steel of a high temper, purposely hardened in order to do the work for which it is used, and we know that gold adheres to the instrument instead of *vice versa*. I venture to say there is not a trace of steel transferred from the instrument to the gold.

Another theory of this discoloration was that which was brought forward by Dr. W. H. Rollins some time ago, that the gold was impure. All the reply that is to be made to that I have already made,—namely, it would be rendered non-cohesive if alloyed sufficiently to cause the amount of discoloration present.

These are all the explanations brought to my notice in regard to the discoloration of gold, of plates or fillings.

I have here a plate which perhaps furnishes a fair sample of the discoloration referred to. A significant fact is that the discoloration of a gold filling is a ruby color, and the discoloration of gold plates is identical with it. This will deepen and assume almost a black color as it accumulates, but it is really a ruby-colored film, contaminated with food, tartar, or tobacco-smoke; of course the color may vary. I will pass this plate around and you will notice the varying shades of color. When held in certain lights,—especially the sunlight,—it is a very deep ruby color; look at it again, and it disappears. The plate was polished by me a few months ago and is not very thickly coated. In different lights it presents varying shades of the same color. The question may be asked, If silver, gold, and copper be alloyed together, will their salts be the same as when produced from the separate metals?

Well, we know that a mechanical union exists between the silver, the gold, and the copper proven in this way. A gold plate alloyed with copper when boiled in nitric acid loses its copper and retains the gold. The union between the metals is therefore simply mechanical, so that the salts would not be changed,—that is, the salts formed will be essentially the salts of the metals possibly intermingled. This fact being settled, let us see what salts of these metals are of a ruby color. First, we will take silver. Are there any ruby salts of silver? It is an easy matter to refer to the standard works in chemistry and ascertain the color of the different salts, and thus make an analysis by exclusion. We find that the chlorides of silver are white, the oxides are brown or black, the sulphides and compounds of sulphides are black. There is a sulphide of silver which is red, but it is a mixture of arsenic and silver. As there is no arsenic or compounds of arsenic in the fluids of the mouth, or in the alloys of gold, this excludes silver.

Now let us examine copper: the sulphides of copper are blue, the chlorides are green, the oxides are blue or brown, the phosphides are black, so in going over the possible salts of these metals that may be formed by metamorphosis in the mouth, there are no salts that could be produced from either of these two metals that would give the ruby color which we find upon the gold of fillings or plates. We have then but one inference left, that it must be a salt of gold itself.

In this analysis by exclusion we have driven the investigation to the last and only metal; all metals that enter into this question are excluded except gold. The oxides of gold are derived from the chlorides,—the only way they can be produced. The chlorides are first produced, using nitro-hydrochloric acid; the oxides and chlorides are both yellow. The sulphides of silver, which form on a spoon when placed in an egg, cannot be produced either upon alloyed gold or pure gold when placed in the same medium for months. I have tried the experiment of placing pure gold and eighteen carat gold in decomposing egg for two months, and they were as bright at the end of that time as when first placed in the test. There remains one salt of gold for us to consider before I call your attention to what I believe to be the true solution of the question,—the purple of Cassius, but as we look at the manner of the formation of purple of Cassius we find that that must be excluded because tin is used with heat, and I do not know that there is any tin used in our gold plates; besides, there is not sufficient heat to develop the color if tin were present. There is no tin used in gold fillings.

We have then circumscribed the field of investigation to one salt of gold, and that is, the phosphate of gold. Some time ago I presented a paper to this society on the decomposition of phosphates in the mouth, the result of some experiments performed in the chemical laboratory of the Harvard Medical School with Professor Hills. These experiments were made to prove the decomposition of the phosphates of the tooth developing free phosphoric acid, and proved the presence of free phosphoric acid in dental caries. These phosphate salts of lime, etc., are not only in the cavities of decay, but around the necks of the teeth and in the fluids of the mouth, a fact well known. The manner of formation or decomposition can alone be a matter of any doubt. In Bloxam, page 261, after speaking upon phosphorus, its action not only upon gold, but upon platinum, this experiment is given, which proves that the combination of gold and phosphorus will produce the discoloration referred to. "By floating very minute scales of ordinary phosphorus upon a dilute solution of chloride of gold, the metal will be reduced in the form of an extremely thin film, which may be raised upon a glass plate, and will be found to have various shades of green and violet by transmitted light, dependent upon its thickness, while its thickest part exhibits the ordinary color of the metal to reflected light. By heating the films on the plate, various shades of amethyst and ruby are developed. If a very dilute solution of the chloride of gold in distilled water be placed in a perfectly clean bottle, and a few drops of ether in which phosphorus has been dissolved, poured into it, a beautiful ruby-colored liquid is obtained, the color of which is due to metallic gold in an extremely finely-divided state, and on allowing it to stand for some months the metal subsides as a purple powder, leaving the liquid colorless. If any saline impurity be present in the gold solution, the color of the reduced gold will be amethyst or blue. These experiments (Faraday) illustrate very strikingly the use of gold for imparting ruby and purple tints to glass and the glaze of porcelain."

There is also another reference in Bloxam, page 434, on this same subject: "If very finely-divided gold be suspended in water, it imparts a violet or red color to it. Such colored fluids, containing very minute particles of gold in a state of suspension, may be obtained by the action of phosphorus dissolved in ether upon a very weak solution of gold in aqua regia."

Although this does not prove conclusively that this color is due to phosphorus, yet there is this point that may be made: That a combination of finely-divided gold and phosphorus produces this color, which is present both upon the pure gold of fillings and upon gold plates. Some days ago one of the physicians of this city was in my office, and I was speaking to him upon this matter, and said that, according to my observations, the discoloration of gold in the mouth was most persistently present in cases of patients of nervous prostration or nervous disorders. I have several patients in mind whose fillings have been tarnished, and the majority of them are subject to some nervous trouble. One of the patients wears a plate which has to be polished every few weeks. We know that the chemical properties of nerve-matter are mainly phosphate of lime or phosphate of magnesia, and a nervous disease would naturally excite the elimination of these substances from the nerve-tissues in various forms. I am not prepared to give the exact reaction which brings about the phosphorus and gold combination spoken of, but the presence of both elements in the mouth, coupled with the coloring referred to, renders the theory advanced a very probable fact.

If these observations are worth anything they prove this: That in regard to the discoloration of gold in the mouth it makes very little difference what carat of gold we use for plates, a high carat is quite as likely to discolor as a low carat,—we may use a fourteen carat or a twelve carat without fear of greater discoloration, and this is consistent with my experience in practice.

The plate passed around at the meeting of which this paper was read was thoroughly cleansed from all foreign matter and subjected to the action of strong nitric acid for three weeks. A part of this acid was tested in the usual way with molibdate of ammonia, for phosphates or phosphorus and a strong reaction gave the confirmation of its presence. The other portion on testing gave traces of gold sufficient to confirm its presence. The solubility of gold of the ruby salt in strong nitric acid is of course a matter of doubt.

